# Bovine Foamy Virus: Shared and Unique Molecular Features In Vitro and In Vivo

**DOI:** 10.3390/v11121084

**Published:** 2019-11-21

**Authors:** Magdalena Materniak-Kornas, Juan Tan, Anke Heit-Mondrzyk, Agnes Hotz-Wagenblatt, Martin Löchelt

**Affiliations:** 1 Department of Biochemistry, National Veterinary Research Institute, 24-100 Pulawy, Poland; magdalena.materniak@piwet.pulawy.pl; 2 Key Laboratory of Molecular Microbiology and Technology, Ministry of Education, College of Life Sciences, Nankai University, Tianjin 300071, China; juantan@nankai.edu.cn; 3 German Cancer Research Center DKFZ, Core Facility Omics IT and Data Management, 69120 Heidelberg, Germany; a.heit@dkfz.de (A.H.-M.); hotz-wagenblatt@dkfz.de (A.H.-W.); 4 German Cancer Research Center DKFZ, Program Infection, Inflammation and Cancer, Div. Viral Transformation Mechanisms, 69120 Heidelberg, Germany

**Keywords:** bovine foamy virus, BFV, foamy virus, spuma virus, model system, animal model, animal experiment, miRNA function, gene expression, antiviral host restriction

## Abstract

The retroviral subfamily of *Spumaretrovirinae* consists of five genera of foamy (spuma) viruses (FVs) that are endemic in some mammalian hosts. Closely related species may be susceptible to the same or highly related FVs. FVs are not known to induce overt disease and thus do not pose medical problems to humans and livestock or companion animals. A robust lab animal model is not available or is a lab animal a natural host of a FV. Due to this, research is limited and often focused on the simian FVs with their well-established zoonotic potential. The authors of this review and their groups have conducted several studies on bovine FV (BFV) in the past with the intention of (i) exploring the risk of zoonotic infection via beef and raw cattle products, (ii) studying a co-factorial role of BFV in different cattle diseases with unclear etiology, (iii) exploring unique features of FV molecular biology and replication strategies in non-simian FVs, and (iv) conducting animal studies and functional virology in BFV-infected calves as a model for corresponding studies in primates or small lab animals. These studies gained new insights into FV-host interactions, mechanisms of gene expression, and transcriptional regulation, including miRNA biology, host-directed restriction of FV replication, spread and distribution in the infected animal, and at the population level. The current review attempts to summarize these findings in BFV and tries to connect them to findings from other FVs.

## 1. Introduction

**Figure 1:**
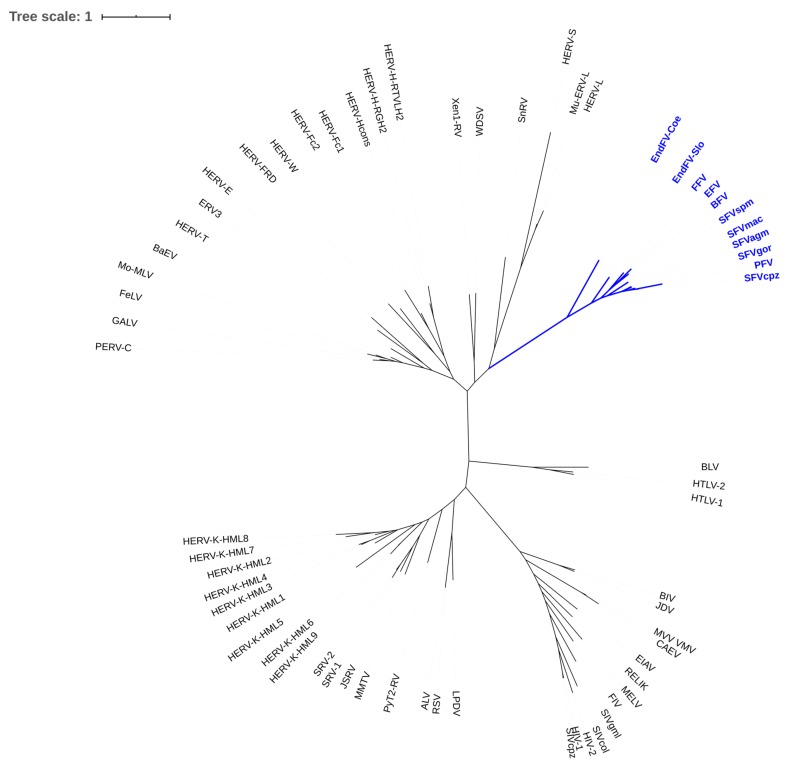
Phylogenetic tree of known exogenous and endogenous foamy viruses (FVs) (blue branches) and members of the *Orthoretrovirinae*. A fasta file with the conserved regions of the Pol proteins (supplement from ref. [2] and prototype FV (PFV, U21247.1) was used for alignment with ClustalW (http://www.clustal.org/). From the alignment, an ML tree was created using fastml (https://fastml.tau.ac.il, default parameters). The resulting newick tree was displayed by Itol (https://itol.embl.de/).

The family of *Retroviridae* is divided into two subfamilies: The *Spumaretrovirinae* consist of five genera of different spuma or foamy viruses with shared and unique features that separate them from the canonical *Orthoretrovirinae*, which comprise all other known exogenous retroviruses (Figure 1) [1]. The number of research groups working on FVs is correspondingly small and even further split by their individual research focus or the FV isolate or host species used in their studies but also due to the sheer difference in numbers and an apparent lack of pathogenicity of foamy viruses (FV).

Most molecular analyses have been conducted on the so-called prototype/primate FV isolate, also initially designated human FV, but subsequently shown to be the end-product of the zoonotic transmission of a chimpanzee FV to an East African naso-pharynx cancer patient [3]. Upon subsequent propagation and passaging in diverse human and non-human cell lines and concomitant severe genetic changes [4], this virus became the best-studied FV isolate and it gained the name prototype FV (PFV). However, its prototypic character might be questioned, since research on highly related simian FVs or more distantly related FVs of feline, bovine, and equine origin is lagging behind and has revealed—at least in selected cases—more or less different data (Figure 1 and Table 1) [5]. Simian FVs share substantial relatedness and, despite having a long co-evolutionary history with their cognate hosts, inter-species transmission is frequent and well documented among closely related hosts, like Old World monkeys and apes, including humans, but also between New World monkeys and humans [5,6,7,8]. In general—and there are only very few exceptions known—FVs co-speciate with their cognate hosts and more or less closely related species may be susceptible to the same or a highly related FV [5,9,10,11]. This host range is likely due to the co-evolution of the virus, together with its host with FVs being the most ancient retrovirus according to the presence of endogenous viruses in all of the vertebrate groups [10,12].

Although a so-called prototype (and/or primate) FV exists in the literature, conserved, prototypic features, besides those basic characteristics that led to the establishment of an independent subfamily of FVs, are currently only partially known. Here, an unbiased comparison of distant FVs and their replication strategies might be worth trying to discriminate basic from deduced, secondary features. In addition, unique data not available for the other FVs have been generated for bovine FV (BFV, see Table 1), and there is the question whether they represent shared or unique features [5]. In this review, we try to use the current data on diverse aspects of the molecular biology of BFV to broaden and complete the overall knowledge of FV biology and indicate avenues of further investigation on BFV biology in vivo and in vitro. In Table 1, the biggest achievements and strengths in the BFV system are summarized and this review will cover some of them in more depth.

## 2. Specific Topics and Highlights in BFV Biology and Virus-Host Interaction

### 2.1. Historic View

**Figure 2:**
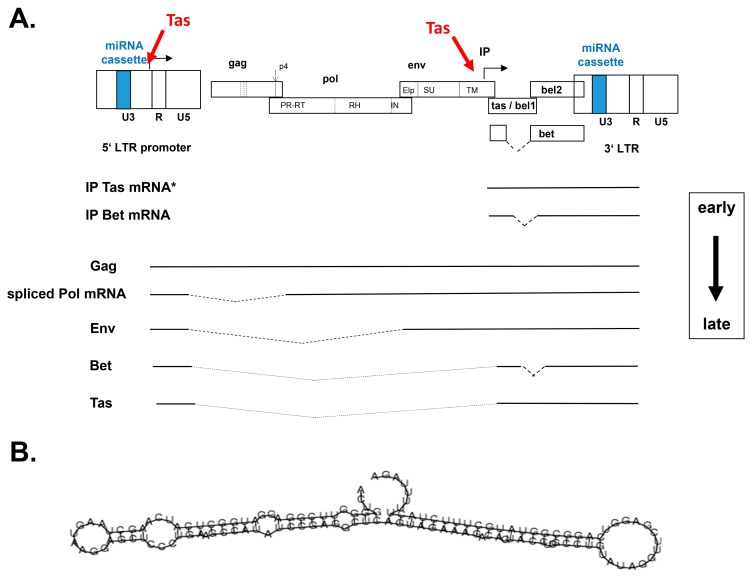
Genetic structure and schematic illustration of bovine foamy virus (BFV) gene expression and the BFV primary miRNA. (**A**) The BFV provirus DNA genome is shown on top schematically and out of scale with the terminal long terminal repeats (LTRs) consisting of the U3, R, and U5 regions. The position of the miRNA cassette in the U3 regions is indicated in color. BFV genes are shown as overlapping open boxes sub-divided into the mature protein domains. Proteolytic processing is marked by dotted lines. The spliced *bet* gene is separately shown below the genome. Broken arrows indicate the transcriptional start sited and direction of LTR- and internal promoter- (IP) directed gene expression and the Tas-mediated transactivation of the 5’LTR and the IP is indicated in red. Below, a selection of the major early and late BFV transcripts starting at the IP and LTR are shown with spliced-out areas indicated by broken lines. Only the major BFV IP-directed Tas mRNA is shown (*). The shift between early and late transcription is marked by a boxed arrow at the right-hand margin. (**B**) The predicted folding and secondary structure of the BFV dumbbell-shaped miRNA precursor (BFV pri-miRNA) is given, for additional information, and the sequence of the mature and stable miRNA, see below and Whisnant et al., 2014 [22].

While the first FV was already described in 1954 [29], the first FV from cattle was isolated 15 years later and designated Bovine Syncytial Virus [30]. The subsequent isolates were also designated Bovine Spuma Virus and Bovine Spumaretrovirus before the name bovine foamy virus (BFV) was coined and finally acknowledged by the ICTV in 1999 (https://talk.ictvonline.org//taxonomy/p/taxonomy-history?taxnode_id=20074661) [1]. Holzschu et al. [19] published the first full-length nucleotide sequence of a BFV isolate from the United States (US) in 1998. These data confirmed the overall genetic structure and coding capacity of BFV as a typical member of the FV genus (Figure 2A).

**Figure 3:**
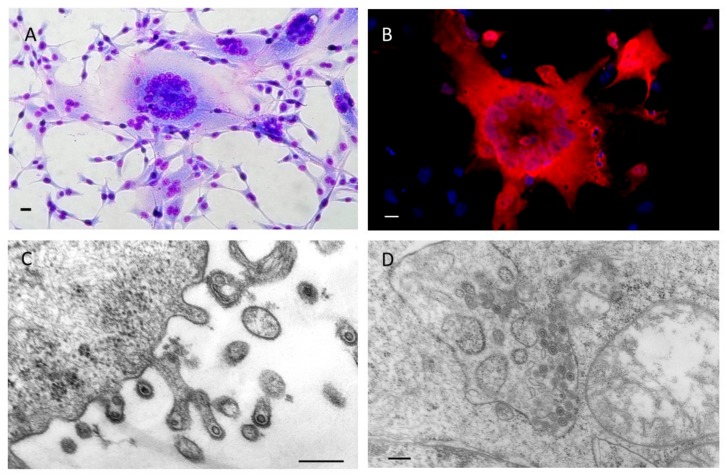
BFV100-infected canine fetal thymus Cf2Th cells: (**A**) Giemsa stained syncytia; (**B**) detection of BFV Gag proteins (red) by indirect immunofluorescence, nuclei were stained in blue; BFV particles budding from the (**C**) plasma membrane (magnification is 60,000-fold) and (**D**) accumulating intracellularly in the endoplasmic reticulum (magnification is 32,000-fold) as visualized by transmission electron microscopy. Scale bars in (**A**,**B**) are 250 µm and in (**C**,**D**) 500 nm.

This opened the way for functional and genetic studies on the molecular biology and replication of BFV in cell cultures and experimentally BFV-infected animals. In addition, it allowed for the establishment of tools for high sensitivity and specificity detection and diagnosis, as described in the subsequent chapters and undertaken in the labs of Jacek Kuzmak and Magdalena Materniak-Kornas, Wentao Qiao, Yunqi Geng and Juan Tan, and Martin Löchelt and co-workers (Figure 3).

Almost unrecognized since exclusively publishing in German, the BFV Riems isolate was established and characterized by Dr. Roland Riebe and co-workers in East Germany (Friedrich Löffler-Institute, Riems, Germany) in the early 80s of the last century [17,18]. The original BFV Riems isolate is, to our knowledge, the only FV that has been exclusively propagated in primary cells of its authentic host species and it thus might have not so much “suffered” genetic changes and co-adaptive imprints due to (repeated) host cell changes and prolonged growth in tumor cells displaying highly selected and aberrant features.

### 2.2. Excellent, Well Established Non-Primate FV Model of Transactivation, Gene Expression and Gene Function

Gene expression and transactivation studies have been mainly conducted in the earlier years of PFV and SFV research, in particular between 1990 and 2000. Research regarding the underlying molecular mechanisms of BFV gene expression has only started in 2008 and it is still ongoing in the lab of Wentao Qiao and Juan Tan while using current, state of the art methods and technologies, thus also extending from this perspective our understanding of FV gene expression and replication as reported here by J.T. (Figure 2A). Similarly, BFV Bet and Gag have been additionally studied by this group during the last years and are thus included in this review, allowing for a more comprehensive view on structural and non-structural FV proteins (Figure 2A).

#### 2.2.1. Function of Tas

Unlike PFV Tas, BFV Tas has no classical nuclear localization signal (NLS), but it is mainly present in the nucleus beside some cytoplasmic localization [31,32,33]. Like most typical DNA-binding transcriptional activators, nuclear localization and multimerization are both required for the transactivation activity of Tas [31,32]. It was reported that PFV Tas has three domains that mediate multimer formation in the nuclei of mammalian cells, but the biological function of PFV Tas multimerization has not been defined [32]. In contrast to PFV Tas, BFV Tas has only one domain that mediates dimer formation. The comparison of the multimerization domains of both proteins does not reveal obvious homologies. Deleting the dimerization region abolishes the Tas-induced transactivation of BFV LTR and internal promoter (IP), which suggests that the active form of BFV Tas is a dimer [31].

There are at least four BFV *tas* mRNAs during BFV infection [34]. These four forms of BFV *tas* mRNA transcripts initiate either at BFV LTR (one) or IP (three), are spliced or unspliced and they have a differential ability to activate the BFV promoters (for clarity, only one representative IP-derived *tas* mRNA is shown in Figure 2A) [34]. According to these findings, we propose the following model of Tas-mediated BFV gene expression. Firstly, activator protein 1 (AP-1) and some other unknown cellular transcriptional genes activate the Tas-mediated transactivation of the BFV IP as the early promoter for BFV gene expression, leading to the transcription of BFV IP *tas* mRNAs [35]. In consequence, BFV Tas quickly accumulates to further enhance BFV IP activity. When a defined threshold level of BFV Tas is reached, the early phase of BFV IP-directed *tas/bet* expression is switched to the late phase of structural gene expression directed by LTR (Figure 2A). The transcription of LTR-spliced BFV *tas* transcripts with low biological activity can ensure modest levels of Tas, which makes it possible to establish persistent viral gene expression to complete the viral life cycle and maintain a balance between the virus and host cells.

Until now, the molecular mechanisms of transcriptional activation by Tas have remained unclear. Past investigations indicate that co-activators p300 and PCAF physically and functionally interact in vivo with PFV Tas, resulting in the enhancement of Tas-dependent transcriptional activation [36]. Subsequently, PCAF acetylation of feline FV (FFV) Tas was shown to augment promoter-binding affinity and virus transcription [37]. Similar to Tas of PFV and FFV, p300 can specifically interact in vivo with BFV Tas, which results in the enhancement of Tas-dependent transcriptional activation [38]. In addition, the p300-mediated acetylation of BFV Tas can increase its DNA binding affinity, and the K66, K109, and K110 residues are critical for the DNA binding ability of BFV Tas; however, they are not conserved among different FVs [38]. The K→R mutations in full-length BFV infectious clones reduce the expression of viral proteins, and the triple mutant completely abrogates viral replication [21]. These findings suggest that acetylation might be an ubiquitous mechanism adopted by FVs as an effective means to regulate gene expression and animal FVs potentially share similarities with PFV in their need for essential host cell factors, e.g., p300 and PACF, etc. In addition to p300, BFV also engages the cellular RelB protein as a co-activator of BFV Tas to enhance its transactivation function [39]. Furthermore, it was found that BFV infection upregulates cellular RelB expression through BFV Tas-induced NF-κB activation [39]. Thus, it is a positive virus-host feedback circuit, in which BFV utilizes the host’s NF-κB pathway through the RelB protein for its efficient transcription [39,40]. There are many other unknown factors that are involved in the transactivation of Tas and some advanced techniques, such as tandem affinity purification and proximity labelling, can be used to discover new co-activators of Tas.

#### 2.2.2. Function of Bet

Although the mechanisms of FV Bet expression by splicing-mediated fusion of the N-terminal domain of Tas to the entire *bel2* coding sequence were described almost 25 years ago, its functions are only partly clarified (Figure 2A) [5,41]. FV Bet is highly expressed after infection by different FVs [13,42]. Previously, FFV and PFV Bet were shown to serve as antagonists of apolipoprotein B mRNA-editing, enzyme-catalytic, polypeptide-like 3 (APOBEC3) family antiretroviral proteins for facilitating PFV and FFV replication [43,44,45]. In addition, Bet might play an important role in the establishment and maintenance of viral persistence in vitro and in vivo [46,47]. Furthermore, Bet has been described as having a negative regulatory effect upon the basal IP activity of PFV and it might limit the expression of the transcriptional transactivator Tas by inhibiting the activation of the IP [48].

BFV Bet consists of 419 aa and it derives from a multiplied spliced mRNA fusing the first N-terminal 35 aa of BFV Tas to the entire Bel2 open reading frame. Although the sequence homology between the Bet proteins of different FVs is very low, some motifs in Bel2 are similar among the different Bet proteins [49]. The Bet proteins of the known BFV isolates [17,19,50,51,52] are highly conserved. In PFV-infected cells, Bet was shown to fuse with Env and form a glycoprotein of ~170 kDa [53], but a corresponding BFV Env–Bet fusion protein could not be detected while using a BFV Bet antiserum.

BFV3026 Bet is present in both the nucleus and cytoplasm (predominantly in the nucleus) of the infected or transfected cells [54]. Analysis of BFV3026 Bet amino acid sequences did not reveal the apparent structural sequence or functional protein motifs, but a nuclear localization signal (NLS) was predicted at the C-terminal end of BFV3026 Bet (392–396 aa) containing the amino acid sequence RRRRR (PSORT II software, [55]) and NLStradamus model, [56]). PFV Bet was reported to have an effective NLS at the C terminus (between 406 and 459 aa), but it does not contribute to nuclear localization of the protein and PFV Bet is located in both the nucleus and cytoplasm [57]. BFV3026 Bet has a similar subcellular localization as PFV Bet, so it will be interesting to determine whether the predicted NLS of BFV Bet is functional. Nuclear pore complexes are known to allow two modes of transport: the passive diffusion of small molecules (<20–40 kDa) and active transport of larger molecules (50 kDa and more) [58]. As BFV Bet (55 kDa) is slightly too large to freely shuttle between the cytoplasm and nucleus, it might enter the nucleus using the NLS or by a currently unknown mechanism.

The functions of Bet during FV infection and replication are seemingly contradictory. It is required for FFV productive replication, as Bet mutants showed approximately 1,000-fold reduced viral titer in feline kidney cells when compared to the wild-type FFV [59]. This is in contrast to PFV, where different Bet mutations or deletions did not show a defined phenotype or only an approximately 10-fold decreased cell-free viral transmission, which suggests that Bet might play a role in efficient cell-free viral transmission [60]. However, these studies were often conducted in heterologous or genetically altered cells. Similarly, the BFV Bet mutant BFV3026 genome showed a four-fold higher level of replication than the wild-type genome in engineered human 293T cells [50]. In addition, similar to PFV [61], the overexpression of BFV Bet in heterologous canine fetal thymus cells (Cf2Th) reduced BFV3026 replication approximately threefold [50]. Taken together, these data suggest that BFV Bet may serve as a negative regulator for BFV replication; however, analyses in authentic host cells appear to be absolutely mandatory.

In summary, these observations indicate that a biologically relevant FV Bet phenotype might only be detectable in cells expressing the cellular partner or target molecules of the authentic FV Bet protein. This is e.g. exemplified by the controversial finding on the Bet-induced inactivation of APOBEC3-mediated virus restriction: if APOBEC3 is either missing in the host cell used or the FV in question is propagated in different host cells without APOBEC3 expression or expression of heterologous APOBEC3 proteins, the intricate interaction between these partner molecules is lost, resulting in irrelevant phenotypes. Similarly, the repeated shifts of FVs from one to another host cell may have had similar consequences. These different scenarios are a strong case to use in vitro homologous host cells without genetic changes and adaptations often occurring in tumor cells or after extended passages in vitro and/or to conduct animal experiments in the authentic host species.

#### 2.2.3. Function and Localization of Gag

The interaction and subsequent self-multimerization of retro- and foamy virus Gag protein cause capsid formation [62,63]. Unlike Gag proteins from Orthoretroviruses, FVs Gag is not processed into separate matrix (MA), capsid (CA), and nucleocapsid (NC) subunits (see Figure 2). In fact, four processing sites have been identified in the PFV Gag protein, which are divided into the optimal C-terminal cleavage site yielding p68/p3 and three suboptimal cleavage sites yielding p33/p39 or p39/p29 [64,65]. In BFV, four Gag cleavage forms (p71, p68, p33, and p29) were also observed, indicating that both the optimal and suboptimal cleavages of Gag protein also occur in BFV; the Gag p68/p3 cleavage is the most efficiently used cleavage site [66]. In contrast to Orthoretroviruses, the C-terminal domain of PFV Gag (the NC domain equivalent) contains three glycine (G) and arginine (R)-rich motifs (GR boxes) or less-defined RG-rich regions instead of the canonical cysteine-histidine repeat motif [67]. Similar to PFV Gag, BFV Gag also has a nuclear location signal (NLS) in GR box II, which causes the nuclear accumulation of overproduced Gag protein [66,68].

Unlike Orthoretroviruses, but similar to the other FVs, BFV Gag is not myristoylated and it cannot produce cell-free Gag-only virus-like particles [24,25]. Similar to hepatitis B virus (HBV), BFV particle budding and release are instead dependent on the co-expression of the cognate viral envelope (Env) protein [24,25], which suggests that Env provides a critical membrane-targeting function inherently lacking in BFV Gag. In the case of BFV, this occurs at the plasma membrane rather than the endoplasmic reticulum (ER), due to a lack of a functional ER retrieval signal (ERRS) [68]. The addition of a membrane targeting signal to the N-terminus of Gag restores Gag-only budding from the plasma membrane, implying that Myr-membrane targeting substitutes for Env in particle release [24,68].

Unlike PFV, FFV, and SFVs, BFV is highly cell-associated and it can only transmit through cell-to-cell but not via cell-free pathways [17,24]. Interestingly, the Gag protein of BFV-Z1 (an in vitro selected cell-free infectious BFV3026 clone) lost a 14-amino acid sequence as compared to BFV-B (an infectious cell-associated BFV clone). This 14-residue deletion is located in the central and non-conserved region of FV Gag, which strongly contributes to the size differences of simian versus non-simian FVs [69]. This deletion led to a smaller Gag-Z1 that enhanced cell-free infectivity by four- to five-fold [25]. At the same site in Gag in some high titer (HT) cell-free BFV-Riems variants, insertions, and duplications occurred. However, their impact on BFV titers has not been studied [26]. The Gag-Env interaction is very important for the budding and release of FV virions. Yet, the interaction of Gag and Env in BFV-B and BFV-Z1 was almost the same, which suggests that the contribution of Gag-Z1 to enhanced cell-free transmission is not through promoting interaction with Env [25].

Viruses must engage bidirectional cellular transport mechanisms for completing their whole life cycle, and many viruses require microtubules (MTs) during cell entry for efficient nuclear targeting or the cytosolic transport of naked viral particles [70,71,72]. In BFV, co-localization of MTs and assembling viral particles was clearly observed in BFV infected cells, which implied that BFV particles or assembly intermediates may transport along the cellular MTs to the cellular membrane to ultimately egress from the host cell. In fact, the MTs-depolymerizing assay indicated that MTs are required for the efficient replication of BFV [66]. In conclusion, BFV has evolved this mechanism to hijack the cellular cytoskeleton for its replication. Until now, it is not clear which components of the MTs are involved in a uni- and/or bidirectional cellular transport of BFV particles. Thus, investigations on the direct interaction between the Gag and MT components should be a future research topic.

### 2.3. BFV-Host Interactions: Restriction Factors, Innate Immunity, miRNAs and Tight Cell Association

#### 2.3.1. Restriction Factors

The innate immune system constitutes a first line of defense against invading viruses. Cellular restriction factors are key players of innate and/or intrinsic immunity, which interferes with defined steps in the viral life cycle, leading to the attenuation or complete suppression of virus replication mainly acting immediately after virus infection [73]. On the other side, viruses have evolved strategies to circumvent this inhibitory activity by co-evolution with host-encoded restriction factors.

Restriction factors are constitutively expressed and their expression can usually be increased by interferons (IFNs) [74,75,76,77]. Until now, several restriction factors acting on retroviruses have been characterized in detail: APOBEC3, tripartite motif protein 5α (TRIM5α), bone marrow stromal cell antigen 2 (BST2, also called tetherin), SAMHD1, IFITM, MxB, and SERINC [78,79,80,81,82,83,84]. Recently, some restriction factors were found to inhibit the replication of FVs. For instance, TRIM5α is implicated in restricting PFV, SFV, and FFV during viral entry [85,86]; APOBEC3 proteins are known to act during PFV, SFV, and FFV reverse transcription (RT), and introduce lethal mutations in the viral genome [43,44,45,87]; whereas, human BST2 (hBST2) and bBST2A1 (one isoform of bovine BST2) suppress the release of PFV and BFV [88,89,90]. Moreover, unlike hBST2, bBST2A1 displays no inhibitory effect on cell-to-cell transmission of PFV and BFV [90]. Other antiviral proteins include promyelocytic leukemia protein (PML), IFN-induced 35-kDa protein (IFP35), N-Myc interactor (Nmi), and p53-induced RING-H2 protein (Pirh2), which have been recently shown to inhibit FV replication through interacting with Tas [27,91,92,93]. PML directly interacts with PFV Tas and it interferes with its ability to bind the TREs in the PFV LTR and IP [91]. IFP35 might inhibit BFV Tas-induced transactivation by interfering with the interaction of a cellular transcriptional activation factor(s) and BFV Tas [27]. Nmi inhibits the Tas transactivation of the PFV LTR and IP by interacting with Tas and sequestering it in the cytoplasm [92]. Pirh2 negatively influences the Tas-dependent transcriptional activation of the PFV LTR and IP by interacting with the transactivator Tas and down-regulating its expression [93]. These antiviral proteins likely limit or modulate the viral spread in vivo, but other antiviral proteins detected, for instance, in a recent high-throughput screen using PFV might, in addition, lead to FV latency, but are currently mostly unexplored [94].

#### 2.3.2. Innate Immunity

An interesting feature of FVs is their ability to infect a diverse range of cell types and cause a characteristic foam-like cytopathic effect in culture system. However, they appear to be non-pathogenic in either naturally or accidentally infected hosts with a currently “emerging” but still ill-defined capacity to affect blood or kidney parameters without overt clinical consequences [11,95,96]. This suggests that the host immune system controls viral infection and/or FV replication in vivo. Some evidence showed that the innate immune system probably plays an important role in limiting FVs replication to superficial epithelial cells of the oral mucosa [97]. It has been suggested in early studies while using human or primate cell lines that FV does not activate an innate response and cannot induce type I IFNs (IFN-I) [98,99,100]. However, only in recent years, it was reported that PFV is efficiently sensed by primary human hematopoietic cells via Toll-like receptor (TLR) 7, which leads to the production of high levels of IFN-I [101]. The PFV-induced IFN-I induces the expression of IFN-stimulated genes in line with the finding that factors restricting FV replication are IFN-I-induced (e.g. TRIM5α and APOBEC3, see above). This activation of the innate immune responses might be a prerequisite for controlling viral replication in zoonotically infected humans or natural animal hosts. In line with this finding, previous studies reported that FV replication is sensitive to IFN-I [98,100,102] due to the induction of several IFN-induced cellular proteins with antiviral activity in culture systems [27,43,89,90,91,92,93]. Besides IFN-I, gamma IFN (IFN-γ) that is produced by activated human peripheral blood lymphocytes has also been found to be a major suppressive factor of PFV [103].

Unfortunately, knowledge regarding the host-cell responses (especially innate immune responses) to BFV infection on the level of gene expression is still limited. In a recent study, changes in the transcriptome of the bovine macrophage cell line BoMac after in vitro BFV infection were examined while using bovine long oligo plus microarray (BLOPlus, Michigan State University, US) technology [104]. In total, 124 genes involved in distinct cellular processes were up- or down-regulated. Among the differentially expressed genes, only five are involved in immune response. Three genes (Hsp90b1, hla-drb1, and Cxorf15) were up-regulated while two genes (CXCL2 and SELENBP1) were down-regulated. However, only the results of all three up-regulated genes (Hsp90b1, hla-drb1, and Cxorf15) were confirmed by subsequent RT-qPCR analyses [104].

The Hsp90b1 protein is essential for the broad tropism of vesicular stomatitis virus (VSV) and for the establishment of infection with VSV and activation of innate immunity via TLRs [105]. Therefore, the Hsp90b1 protein might have an effect on the capacity of FVs to infect a variety of tissues from different organisms. In addition, HLA-DRb1 (major histocompatibility complex class II, DR beta 1), an HLA class II antigen, plays central roles in immunity by presenting peptides derived from foreign, non-self proteins. It was found that specific HLA haplotypes, including HLA-DR, may protect against human immunodeficiency virus (HIV) [106], and MHC class II molecules are up-regulated in several lymphoid cell lines following infection with feline immunodeficiency virus (FIV) [107], as well as in T-lymphocytes of FIV-infected cats [108]. Taken together, the increased level of HLA-DRb1 in BFV-infected BoMac cells might be responsible for the sustained elevation of MHC class II antigen levels. Furthermore, Cxorf15 γ-taxilin, together with α- and β-taxilins, is a member of the taxilin family. β- and γ-taxilin may play a role in intracellular vesicle trafficking [109], and the α-taxilin levels are elevated in hepatitis B virus (HBV)-expressing cells and are essential for the release of HBV particles [110]. One can assume that the upregulation of the Cxorf15 gene following BFV infection suggests a possible role of this protein in virion egress while taking similarities in budding strategies for FVs and HBV into consideration [111]. These data offer a basis for further investigation of the immune response of host cells to FV infection, but the above speculation also needs to be further experimentally confirmed.

The effects of BFV infection on immune gene networks were confirmed in a recent study using experimentally infected calves; however, the differentially expressed genes identified one and three days after infection of the animals were different from those reported for the in vitro study while using BoMac cells [14,104].

#### 2.3.3. miRNA Expression as an Additional Layer to Control Host Gene Expression and Innate Immunity

Recently, BFV and simian FV of African green monkey (SFV_agm_) have been shown to encode miRNAs via RNA polymerase III (RNA Pol III)-directed expression of a complex double-hairpin and, thus, dumbbell-shaped primary miRNA (pri-miRNA) precursor (Figure 2A,B) [22,112]. The identification of such FV miRNA cassettes of about 120 nt length was stimulated and directed by prior findings in bovine leukemia virus (BLV), which is a close relative of human T cell leukemia/lymphotropic viruses (HTLVs) and used as an animal model for its human counterpart [113,114]. The BLV RNA Pol III-driven miRNA cassettes consist of single hairpins structures and they were identified by an algorithm combining the search for RNA Pol III promoters and terminators and the presence of stable RNA hairpin structures that were flanked by these RNA Pol III-specific features [113].

In BFV-Riems, only a single two-hairpin, dumbbell-shaped pri-miRNA with its RNA Pol III promoter and terminator is present in the non-coding part of the LTR U3 region downstream of the *bet/bel2* open reading frame (Figure 2A) [22]. In contrast, in SFV_agm_, several different miRNAs are encoded by either dumbbell-shaped precursors RNA Pol III cassettes and possibly other pri-miRNAs that have been mapped to corresponding sites in the 3’ end of the SFV_agm_ U3 region [112] (see below and Table 2). In both studies, the miRNAs were identified and characterized by miRNA sequencing. In BFV, two high level expressed miRNAs comprising about 70% of the total miRNA pool and a third one at modest levels were detected, a potential fourth miRNA from the remaining strand of the second hairpin was undetectable [22]. In contrast, and reflecting the complexity of situation in SFV_agm_, sequencing identified three high-abundant, two intermediate, and at least six low abundant mature miRNAs [112].

The different miRNA expression capacity and underlying mechanisms of BFV versus SFV_agm_ [22,112] encouraged us to conduct bioinformatics while using the online available and further optimized algorithms to study the situation in BFV-Riems, SFV_agm_, and other FVs. By modifying the original algorithm of Kincaid et al. [112] we especially focused on dumbbell structures of about 130 nucleotides in size in the LTR sequences of 38 FVs (Table 2). Kincaid et al. also analyzed most of them for miRNA structures (Table S1 in [112]). We predicted for 37 of the 38 FV sequences one or more dumbbell miRNA structures while using a fold energy cutoff of −30 kcal/mol and the existence of a terminator together with a TATA- and/or A/B-Box (as overview, see Table 2).

We confirmed the presence of a single miRNA cassette encoding a dumbbell-shaped pri-miRNA [22] in the genome of all known BFV isolates by using the updated algorithm (Table 2). Single dumbbell-shaped pri-miRNAs were also predicted for the closely related EFV and several SFVs from different simian hosts as well PFV derived upon zoonotic transmission into humans. Surprisingly, while a single dumbbell-shaped miRNA cassette was detected in SFV_gor_, it was absent in another SFV_gor_ sequence that was derived from a zoonotically infected person [115]. Our algorithm found each two independent RNA Pol III dumbbell-type pri-miRNA cassettes in SFV_agm_ (representing S1/S2 and S6/S7 miRNAs in [112]). For other SFVs, two, three, and even five dumbbell-shaped miRNA cassettes were predicted. While the different FFV isolates from domestic cats contained four miRNA cassettes, the highly related FFV variant that was derived from Puma concolor was predicted to only encode three miRNAs.

In general, the predicted dumbbell miRNA cassettes are located in the non-coding region of the U3 LTR sequence, except the first miRNA cassette of all FFV isolates, which partially overlaps the 3’ end of the *bel2/bet* gene. In addition, the fourth miRNA cassette of the domestic cat FFVs is very close to the transcriptional start site of the LTR promoter and it might interfere with RNA Pol II-directed mRNA expression similar to the situation in SFV_Cni_, where the third miRNA cassette even extends into the R region (Table 2). The size of the predicted dumbbell-shaped pri-miRNA of the different FVs varies between 111 and 128 nt.

As experimental miRNA sequencing data are currently only available for SFV_agm_ and BFV-Riems, it is currently an open question as to whether these bioinformatics-based predictions presented here properly reflect the expression capacity and strategy of the different FVs and whether there is a huge variability of miRNAs between different, and even closely related, FVs. Additionally, the experimentally detected central SFV_agm_ miRNA and the corresponding stem-loops 3, 4, and 5 [112] were not detected by our dumbbell-specific miRNA detection tool, so that, in certain FVs, there may be a co-existence of single-hairpin and dumbbell-shaped pri-miRNAs. Alternatively, the central SFV_agm_ stem-loops 3, 4, and 5 may be derived from larger, more complex pri-miRNAs, for instance, with terminal stem-loops but separated by unfolded, single-stranded intervening sequences.

The two independent experimental studies [22,112] and our in silico analyses show that probably all FVs of different origin contain at least one RNA Pol III-directed miRNA cassettes of the dumbbell-shaped type. The miRNA repertoire of in SFV_agm_ is clearly more complex than that of BFV and it is currently unknown as to whether other FVs may or may not encode also SFV_agm_/BLV-like single hairpin pri-miRNAs. Thus, further wet biology analyses, high throughput sequencing and bioinformatics are needed to allow for full understanding of this highly important regulatory system of FVs.

The importance of cellular miRNA processing factors dicer and drosha was shown for SFV_agm_ [112], while, for BFV, the impact of the overall shape of the dumbbell-shaped pri-miRNA was demonstrated [22]. In BFV, minor modifications of the pri-miRNA sequence are well tolerated, while the replacement of an authentic stem-loop by heterologous shRNA sequences reduced but did not eliminate reporter gene suppression in dual luciferase assays [23]. This finding, together with high-throughput optimization procedures, as done, for instance, for the BLV RNA Pol III miRNA cassettes [116], may open the way to engineer efficient and specific chimeric FV-based pri-miRNA constructs for therapeutic application, as discussed below (see below, Section 2.5).

Similarities to host miRNAs were detected for experimentally validated SFV_agm_ and BFV miRNAs [112,117] (see Table 3). SFV_agm_ miR-S4-3p shares seed identity and functionality with host miR-155, a noted host oncogenic miRNA (oncomiR). SFV_agm_ miR-S6-3p shares seed identity with the host miRNA family miR-132, which suppresses innate immunity. In contrast, the similarities that were detected for BFV miRNAs comprise the miRNAs bta-miR-125a and the human counterpart of this miRNA has been described as stabilizing the suppressive phenotype of R848-stimulated antigen presenting cells on different levels in a hsa-miR-99b/let-7e/miR-125a cluster [118]. Furthermore, miR-125a and miR-125b are both involved in the progression of cervical cancer [119]. MiR-125b inhibits the PI3K/AKT pathway through the down-regulation of mRNA and protein PIK3CD, while miR-125a is anti-oncogenic by the downregulation of TRIB2 and HOXA1 by the family miR-99 clustered in miR-let-7c~99a, miR-125a~let-7e~99b, and miR-100~let-7a-2. The members of these clusters are diminished in cervical cancer [120]. Taking all of this together, the miRNAs coded by FVs seem to interfere with immune and proliferation processes in the cells, but in different ways. The transcription by RNA Pol III makes the expression of miRNAs independent from protein expression, while the location in the LTR avoids the restrictions that are imposed by overlapping coding regions, thus enhancing variability and adaptability.

In BFV, three different and closely spaced miRNAs are detectable in chronically or lytically infected cell culture cells and, importantly, also in experimentally BFV-infect calve peripheral blood mononuclear cells (PBMCs), with the latter confirming the relevance of these findings beyond cell culture systems [22]. The three stable miRNAs are generated from both stem-loops of the dumbbell-shaped pri-miRNA [22]. In chronically BFV-infected cells in vitro, two BFV miRNAs make up more than 2/3 of the total cellular miRNA pool pointing to an important role, especially in chronically infected cells [22]. The two high-abundance miRNAs are localized each to the 5’part of the two different stem-loops, while in SFV_agm_, miRNAs from both strands of the S3 stem-loop are easily detectable [22].

In the BFV system, bovine cells and bovine genomics have been used and the outcomes of bioinformatics-guided target gene prediction as well as wet biology-based experimental validation were mostly conducted in bovine cell cultures and finally in BFV-infected cells and experimentally infected cattle, as described by Cao et al. [14]. In brief, target site predictions for the high abundance BF2-5p miRNA yielded several potential targets with very high scores and two of them with relevance for innate immunity and virus replication, ANKDR17 [121] and Bif-1 (SH3GLB1) [122] were experimentally confirmed in independent assays [14]. In addition, even downstream targets of ANKRD17 showed altered expression in response to BFV miRNA cassette deletion and miRNA co-transfection [14]. A small number of calves were infected with MDBK cells expressing the highly cell-associated BFV Riems isolate and high-titer in vitro-selected BFV variants lacking or carrying the miRNA cassette in order to establish conditions for in depth analyses of the importance of the miRNAs in the authentic host [14]. The data show that all BFV variants are replication-competent in calves; however, the deletion of the miRNA cassette caused a drop of viral infectivity. The deletion of miR-BF2-5p probably reduced the replication competence of the virus, as seen by the lower induction of genes involved in the recognition of viruses by the innate immune system when compared to wt BFV-infected calves. It probably also resulted in the lower level of the humoral response to BFV Gag observed, especially in one of the animal infected with the miR-BF2-5p-deficient BFV variant (for details, see [14]).

#### 2.3.4. Highly Cell-Associated Spread, at Least in Cell Cultures—What Is Behind This Phenotype?

Viruses have two major transmission strategies: cell-free transmission, involving the release of virus particles into the extracellular space, and cell-to-cell transmission [123]. Retroviruses exhibit different degrees of cell-free and cell-to-cell transmission. Unlike most other retroviruses, such as HIV-1, murine leukemia virus (MLV), PFV, FFV, and SFV, which transmit through both cell-to-cell and cell-free pathways, the transmission of BFV is highly cell-associated, with very low to undetectable cell-free transmission [17,24]. This lack, or only low level, of cell-free transmission appears to be independent of whether the BFV isolates have been exclusively propagated in primary bovine cells (the BFV Riems isolate) or whether immortalized bovine (MDBK cells) and hamster and canine cell lines, like BHK-21 and Cf2Th, have been used for virus propagation. BFV is an excellent model for studying virus adaption to cell-free transmission and identifying the principles of viral transmission by in vitro selection and evolution analyses, since the BFV particle budding machinery is similar to that of other FVs [24,25].

In two independent selections screen using established immortal MDBK and BHK-21 cells, BFV Riems was shown to adapt to cell-free transmission within 80 and 130 cell-free passages reaching titers of more than 10^5^ and 10^6^ FFU/mL, respectively. The resultant HT variants had independently gained the capacity to spread via cell-free particles, but still also use cell-cell transmission [24]. Genetic studies indicate that consistent and cell type-specific, as well as cell-type-independent adaptive changes, occurred in Gag and Env as well as in the LTR regions where larger changes had also been observed [26] (Bao, Stricker, Hotz-Wagenblatt, and Löchelt, to be published). Importantly, cell-free HT BFV-Riems is still neutralized by serum from naturally infected cows [24]. The different selected HT BFV variants will shed light into virus transmission and the potential routes of intervention in the spread of viral infections.

Zhang and colleagues successfully isolated HT cell-free BFV strains from the original cell-to-cell transmissible BFV3026 strain (Chinese isolate) while using in vitro virus evolution and further constructed an infectious cell-free BFV clone, called pBS-BFV-Z1, to independently explore the molecular mechanisms of BFV cell-free transmission [25]. Following sequence comparisons with a cell-associated clone pBS-BFV-B [50], a number of changes in the genome of pBS-BFV-Z1 were identified. Extensive mutagenesis analyses revealed that the C-terminus of Env, especially the K898 residue, controls BFV cell-free transmission by enhancing cell-free virus entry [25]. The authors also claim that virus release of this variant is increased, although this was not experimentally analyzed [25]. It is well-known that lysine (K) can undergo methylation, acetylation, succinylation, ubiquitination, and other modifications, which play an important role in regulating the protein activity and structure adjustment [124]. Interestingly, the equivalent position of the 898 residue in all known BFV isolates (from the United States, NC001831.1. [19], China, AY134750.1 [50], Poland, JX307861 [51,52], and Germany JX307862.1 [17,52], which only spread through cell-to-cell, is not a lysine (K), while the equivalent position is occupied by a lysine in other high titer cell-free FVs, such as SFV, FFV, and PFV. This suggests that the K898 in Env has an important role in FV cell-free transmission. The underlying mechanisms warrant further studies. Interestingly, the Gag protein of BFV-Z1 lost 14 amino acids in the highly divergent sequence between the matrix and capsid regions, which enhanced cell-free infectivity by four- to five-fold [25]. Other changes of the BFV-Z1 genome contributed little to BFV cell-free transmission. Taken together, these data reveal the genetic determinants that regulate cell-to-cell and cell-free transmission of BFV, and suggest the possibility of generating high-titer BFV vectors through engineering viral Env and, in particular, its C-terminal sequence.

### 2.4. BFV-Host Interactions at the Organismal and Populational Level

#### 2.4.1. BFV Epidemiology and Naturally Occurring Co-Infections

Infections with BFV have been reported worldwide since the first isolation of BFV by Malmquist and co-workers in 1969 [30]; however, sero-epidemiological data are only available from some countries and they show variable rates of BFV infected animals. The highest sero-prevalence was reported in Canada, where it varied between 40 and 50% [125,126]. A slightly lower rate of 39% was observed in Great Britain [127] and Australia [128]. The most recent data come from Germany, where only 7% of tested animals were identified as being BFV positive [15] and from Poland with BFV sero-prevalence of over 30% in dairy cattle [129]. BFV prevalence based on these studies seems to be very diverse. However, these data span a long time-frame; therefore, one of the reasons for these disparities might be the different sensitivity of methods used for serological testing, from agarose gel immuno-diffusion (AGID) and syncytia inhibition assay to ELISA and indirect immuno-fluorescence assays. Additionally, the age of animals tested might be a reason of such diverse BFV prevalence, but, although the age of the animals was not specified in these studies, the importance of this factor was suggested by Jacobs and co-workers, who observed a higher rate of BFV positive status in older animals [126]. This might be due to the persistence of BFV infections and prolonged sero-conversion in animals, but one cannot exclude other factors, like breed and the type of animal rearing. Interestingly, no disease or clear clinical symptoms were ever associated with BFV infection in cows. However a role of BFV as a co-factor of other retroviral infection has been suggested, especially in the context of mixed infections, which are one of the characteristic features of retroviral infections [130]. This has been also suggested for people infected with HIV and HTLV [131], cats infected with FIV, FeLV, and FFV [132], FIV/FeLV [133], and FIV/FFV [134], and monkeys infected with SIV and STLV [135]. Similar studies have been carried out with respect to co-infections with BFV, bovine leukemia virus (BLV), and bovine immunodeficiency virus (BIV) in cattle. Amborski and co-workers published the first report on BFV co-infection with other lymphotropic retroviruses in dairy cows [136]. In the already quoted study by Jacobs and others carried out in Canada, including numerous dairy cattle herds, it was shown that the percentage of animals with antibodies to BIV, BLV, and BFV is 5.5%, 25.7%, and 39.6%, respectively, however with no statistically significant correlation between the individual values [126].

It is assumed that the source of mixed infections might be due to the same route of retroviral transmission, which results in a statistically significant correlation in the occurrence of antibodies, e.g. for BLV and BIV [137] or FIV and FeLV [138,139] and HTLV II and HIV [140]. Mixed infections are particularly important in herds with BLV-infected animals, due to the fact that BLV is the etiological agent of enzootic bovine leukosis and since BFV has been suggested to act as a cofactor in BLV infections [130]. In a recent study, a statistically significant correlation between the occurrence of serologically positive reactions for BLV and BFV at the herd level was shown [141]. Although the results of these studies cannot be related to individual animals, they indicate a certain pattern in the distribution of herds, where BLV and BFV are present. In the study by Jacobs and others, mixed infections of BLV and BFV were recorded in 9.9% of cows [126]. It has been suggested that BFV and BLV co-infections may impair the immune defense capacity of the host [136,142], similarly as proposed for cats co-infected with FIV and FFV [143]. However, it might also be considered that both viruses interact at the molecular level, especially since both of them use the phenomenon of transactivation in the process of viral replication and, even more interesting, they encode miRNAs that interact with genes directly involved in immune defense processes of the host. There is evidence that BLV- and BFV-encoded miRNAs target genes involved in innate and adaptive immunity and, thus, dysregulating their expression levels might facilitate BFV spread, transmission, or persistence. [14,144,145].

BFV is endemic at high prevalence in livestock cattle in different parts of the world, which can be quite easily confirmed by virus isolation in different types of cells (Cf2Th, BoMac, MDBK, KTR, BHK21); however, it is also possible to develop productive infection under experimental in vivo conditions. Only few reports confirmed the possibility of the experimental infection of cattle with BFV [128]. Materniak and co-workers used the experimental BFV inoculation to determine its replication and immunogenicity, not only in its homologous, but also in the heterologous host [13]. Calves and sheep were selected to analyze the infection kinetics in different, but related, species. Although, neither the experimental BFV infection of calves nor sheep resulted in pathology, BFV spread and replicated to similar degrees in both, the homologous and heterologous hosts. Productive BFV infections were established in calves and sheep, as confirmed by virus isolation from leukocytes of all infected animals. BFV was rescued from both infected animal hosts, even in the presence of BFV-specific antibodies, confirming that BFV infection is not cleared by the host immune system [13]. Additional parameters of BFV infection, like humoral immune response to BFV proteins and the presence of BFV DNA in blood cells and organs, also confirmed the persistence of BFV in both hosts [13]. Interestingly, upon long-term replication in sheep, approximately 70% and 40% of the single nucleotide mutations in the sheep-derived BFV *bet* and *env* sequences, respectively, led to changes in the amino acid sequence. As no consistent pattern of adaptive changes was detectable, this proves the utility of sheep as an animal model to study the biology of persistent spumavirus infections [13].

#### 2.4.2. BFV Transmission Route

The transmission of BFV is suggested to occur through close contact. While considering cattle behavior, it is assumed that BFV shedding occurs via saliva through non-aggressive contact, like sneezing or licking and via infected milk [143]. The successful recovery of BFV from saliva and milk of naturally infected cattle tends to confirm this mode of virus shedding and transmission [15,16]. Interestingly, older studies that were reported by Johnson and others, as well as Kertayadnya et al., showed that calves being BFV negative at birth or the beginning of the experiment became infected when placed together with infected adults [128,146]. Additionally, Johnson and others studied different routes of transmission using BFV infected culture fluids containing cell debris. This experiment showed that only throat spray and intravenous application resulted in successful BFV infection in calves, while the swabbing of cell culture-derived BFV into the vagina or onto the prepuce did not lead to infection [128]. Kertayadnya and others also excluded insect or airborne transfer of BFV infection [146]. The authors of both reports state that the most possible source of infection under natural conditions is close contact to a single immunologically tolerant individual, which is productively BFV-infected, but has no or only very low levels of BFV-specific antibodies. However, the source of such BFV tolerance is disputable. One hypothesis is that such animals are characterized by a very early viremic stage of infection before the development of neutralizing antibodies occurs. However, as Kertayadnya and others reported, there are animals that are productively infected with BFV, but do not produce antibodies, even after several months of infection [146]. Another explanation of immunological tolerance towards BFV could be via in utero infection. Such a scenario might be supported by the studies of Bouillant, and Ruckerbauer who recovered BFV from the uterus of BFV infected cows [147]. Finally, perinatal transmission of BFV via colostrum or milk has been proposed. This route of BFV spread is supported by our studies showing that BFV can be reproducibly isolated from the cellular fraction of raw milk [15,16].

#### 2.4.3. Interspecies and Zoonotic Transmission of BFV as Part of the Human Food Chain

It has been clearly demonstrated that SFVs can be zoonotically transmitted to humans in Africa, South America, and Asia upon exposure or contact with SFV-infected monkeys [6,8,148]. The nature of human exposure to BFV is slightly different, but it seems to be constantly present in the human food chain when considering the routes of virus transmission and replication sites of BFV. Additionally, there are many products of cattle origin used in pharmaceutical and cosmetic industry. However, direct contact, which seems to be the most likely mode of zoonotic transmission, is restricted to a limited part of the human population. So far, two serological studies were performed to screen for BFV in humans. One of them focused on dairy cow caretakers, cattle owners, and veterinarians who were tested for BFV-antibodies and showed an overall sero-prevalence of about 7%; however, none of them was PCR positive for BFV DNA in PBMCs [149]. Another study included three groups of humans, who were serologically tested for BFV antibodies [150]. BFV-specific reaction was found in 7% of immunosuppressed patients, 38% of people claiming contact with cattle, and 2% of the general population with no interaction with cows. In each group, a single BFV PCR positive individual was identified and the sequence of short PCR product showed high homology to BFV isolates that are available in GenBank. The data obtained suggest that BFV zoonotic infection may be possible, however it is not common and, in most of the cases, cleared by the host immune system.

#### 2.4.4. BFV Replication in Naturally and Experimentally Infected Animals

In vivo studies play a vital role in virology, since they allow for investigation of the events taking place during the viral infection in the host. Many aspects of infection can be, in fact, only explored by examining naturally infected animals; however, this needs to be done under carefully controlled conditions in the homologous or a heterologous host. In studies on the replication of BFV in vivo, both directions were used; therefore, these data are quite comprehensive. Similar to other FVs, BFV shows a wide tissue tropism. In naturally infected animals, BFV was recovered from peripheral blood leukocytes/lymphocytes, tumors, fetal tissues, placenta, testis, and from fluids used to flush the uterus and oviducts of super-ovulated cows [17,30,51,151,152,153,154]. Studies on naturally and experimentally infected animals using PCR-based virus detection showed that BFV DNA is present in most tissues, like lung, salivary glands, liver, spleen, and bone marrow [13]. Interestingly, some reports from SFVs in monkeys previously showed that, although DNA is present in most animal tissues, SFV RNA, indicative of viral gene expression and replication, is mostly if not exclusively detected in oropharyngeal sites [155,156,157]. In the most recent studies on tissue distribution of BFV DNA and RNA, different organs, as well as blood, bronchoalveolar lavage cells (BALs), and trachea and pharynx epithelium of experimentally infected calves, were analyzed [28]. The highest load of BFV RNA was detected in the lungs, spleen, liver, PBMC, BALs, and trachea epithelium, while in contrast to the previous studies showing BFV isolation from saliva and milk cells of naturally infected cows [15,16], BFV RNA was detected in the saliva of only a single calf. The presence of BFV RNA in such diverse organs seems to be strong proof that active replication of BFV might be not limited to the oral cavity, in contrast to the findings for SFV gene expression in monkeys.

In a recent study, HT BFV Riems, the cell free variant of BFV, and wild type BFV Riems isolate were used for the experimental inoculation of calves [14,24]. The infection pattern was very similar in both groups of calves. The humoral response was comparable in both groups, but BFV viral load measured in PBMCs of infected animals during 16 weeks p.i. was slightly lower in calves that were infected with HT-BFV Riems. However, at the end of experiment, BFV was rescued from PBLs of all, parental and HT BFV-infected, animals. Interestingly, when changes in the expression of selected genes involved in innate immunity were analyzed at day 1 and 3 p.i., the level of induction was clearly lower in the HT-BFV Riems infected calves as compared to wt BFV Riems inoculated animals, which suggests a slightly impaired detection of HT BFV.

Importantly, and confirming the concept that interspecies transmission of FVs is possible to genetically related hosts, sheep have been shown to be permissive to experimental BFV infection while using intravenous inoculation of BFV100-infected Cf2Th cells [13]. Successful inoculation of sheep with BFV as well as its transmission via saliva may support the risk of cross-species infections in mixed farms, where cattle and sheep are kept in close contact. Previous work, in fact, reported the presence of an FV-like virus in sheep [158], but two scenarios are possible since no further characterization of the isolate was performed. One is that the infection was a result of a cross-species transmission of BFV from cattle; alternatively, the isolate was, in fact, a sheep specific foamy virus. Over 500 German sheep serum samples were tested to try to answer this question and 35 sheep sera showed reactivity to BFV Gag antigen in GST ELISA [149]. Unfortunately, further diagnostics of these BFV-cross-reactive animals by virus isolation or PCR amplification were unsuccessful [159]. Recent studies regarding wild ruminants revealed a similar scenario: some sera also reacted with BFV antigens in ELISA test, but PCR mostly failed to identify genetic material of BFV [160]. In fact, the lack of amplification with BFV-specific primers might suggest the infection with novel, species-specific FVs, which generate antibodies that cross-react with BFV-specific antigen. Therefore, the existence of BFV-related FVs in other ruminants still remains open.

### 2.5. Utilization of BFV as Viral Vector for Translational Applications

As with other retroviruses, different viral vectors for the expression of therapeutic genes or the delivery of vaccine antigens have been constructed for PFV, few SFVs, and FFV, as review see [161]. These vectors mostly include replication-deficient gene transfer vectors generated in producer cells, but they also include replication-competent engineered viruses, which are mostly intended for life vaccine applications. Some of these vectors have been tested beyond cell cultures in small lab animals (mostly mice), but also in outbred hosts like dog (ex vivo PFV-based gene transfer vectors to treat canine leukocyte adhesion deficiency [162]) and cats (FFV-based replication-competent vaccine vectors [163,164]). The recent cloning of full-length BFV genomes that allows for cell-free transmission [14,25,26] is an important prerequisite for conducting and extending corresponding studies also for BFV.

Cattle are important livestock animals and vector-based approaches are likely to meet the costs imposed by bovine infectious disease or the need to engineer defined traits in the future. Such vector-directed gene transfer and vaccination might be an interesting option with a corresponding market to explore and use BFV as a suited viral vector for treatment of cattle. The availability of CMV-IE-driven BFV genomes and recent data that HT cell-free BFV variants replicate in cattle are important prerequisites for such studies [14]. Furthermore, even an engineered HT cell-free BFV variant lacking the entire miRNA cassette replicated in experimentally infected animals and induced immunity against BFV Gag and Bet [14]. Together with the data on the function and core features of the BFV miRNA cassette and the chimerization of the BFV pri-miRNA [23], the insertion of therapeutic or prophylactic miRNAs into replication-competent BFV vectors or the construction of BFV-based miRNA expression tools appear to be new and interesting future developments [161].

## 3. Conclusions and Outlook

As shown here, research regarding diverse aspects of BFV replication and biology in vitro and in vivo has significantly expanded our understanding of the complexity and diversity of FVs. These new findings are in line with the concept that each of the known exogenous FVs has been shaped by a long history of co-adaptation and co-evolution [10]. It remains to be seen whether, for instance, the host dictates the major pathway of FV transmission: Here, strong differences between herbivorous cattle that do not display aggressive intra-species behavior and carni- and omnivorous simians or felines with a substantial amount of aggressive behavior within and between groups and individual animals can be anticipated. Whether such differences in the host’s biology not only affect transmission, but also the repertoire of antiviral restriction, remains to be seen, but appears to be possible.

Additional avenues of high-impact research in BFV may be related to development of vaccine vectors based on the BFV genome or genetic elements thereof. The possibility of cell-free BFV infections recently achieved offset the limitations of a tight cell-associated transmission from BFV as a therapeutic and prophylactic vector candidate [14,24,25]. Vaccines that are based on bovine virus-based vectors could be a great alternative in veterinary science and practice, especially in the context of economically important infections that, due to the high prevalence in cattle populations, cannot be eradicated by culling. Furthermore, extending the studies on the BFV miRNAs as modulators of the virus-host interface and evolutionary struggle, as well as their translational application within a BFV-based vector or as an independent cassette, appears to be a promising extension of ongoing work. Finally, it is of high priority to explore the “requirements” of BFV—as part of the human food chain and present in several raw cattle products—to enter the human population. Such studies may be pretty challenging and also—to a certain degree—unpredictable, but of high medical and epidemiological importance. Here, in vitro selection and evolution screens employing either fresh animal-derived BFV or “native” BFV isolates and primary human cells of different tissue/organ origin will be of special value.

## Figures and Tables

**Table 1:** Special features and novel insights that were gained from past and current work on Bovine Foamy Virus.

Subject/Topic	References
BFV as a well-established infection model in life-stock animals (cattle and sheep)	[13,14]
BFV as the only known FV in the general human food chain (beef and dairy products)	[15,16]
BFV Riems as the only FV passaged exclusively on primary and homologous host cells	[17,18]
Integrase domain: disrupted HH-CC zinc finger and unique sequence insertion into the extreme C-terminus	[19]
Detailed understanding of gene expression and transactivation of a non-simian FV	[20,21]
RNA Pol III miRNAs, unique precursor structure and their functions	[14,22,23]
Extremely tight cell association and identification of residues critical for this phenotype	[24,25,26]
Detailed understanding of new restriction factors against FVs	[27]
Broad tissue tropism and gene expression in BFV-infected calves	[5,28]

**Table 2:** Results of bioinformatics on dumbbell-type RNA Pol III cassettes in the LTRs of selected FVs flanked by consensus TATA boxes and termination signals.

Virus-Type	Virus Isolate * and Accession Number	Number of Dumbbell-Shaped miRNA Cassettes	Number of AB or BB Boxes
BFV	BFV_Riems [22]; JX307862.1	1	0
	BFV_100; JX307861.1	1	0
	BFV_11; U94514.1	1	0
	BFV_3026; AY134750.1	1	0
EFV	EFV; AF201902.1	1	0
FFV	FFV Chatul-3; AJ564746.1	4	4
	FFV F17; U85043.1	4	4
	FFV FUV; NC_039242.1	4	4
	FFV_Pco_; KC292054.1	3	3
HFV	HFV; U21247.1	1	0
	HSRV1; Y07723.1	1	0
	HSRV2; Y07724.1	1	0
	PFV; Y07725.1	1	0
SFV	SFV_AG15; JQ867462.1	1	0
	SFV_agm_; NC_010820.1 [112]	2	1
	SFV_AXX; EU010385.1	5	3
	SFV_BAD327; JQ867463.1	1	0
	SFV_BAD468; JQ867465.1	1	0
	SFV_BAK74; JQ867464.1	0	0
	SFV_CAE_FV2014; MF582544.1	2	1
	SFV_CAE_LK3; M74895.1	2	1
	SFV_CJA; GU356395.1	1	0
	SFV_CNI; JQ867466.1	3	1
	SFV_CPZ; U04327.1	1	0
	SFV_GOR; HM245790.1	1	0
	SFV_MAC; X54482.1	1	0
	SFV_MCY; KF026286.1	1	0
	SFV_MFA; LC094267.1	1	0
	SFV_MFU; AB923518.1	1	0
	SFV_MMU; MF280817.1	1	0
	SFV_OCR; KM233624.1	1	0
	SFV_ORA; NC_039085.1	2	1
	SFV_PPY; AJ544579.1	3	2
	SFV_PSC; KX087159	1	0
	SFV_PVE; NC_001364.1	1	0
	SFV_SSC; GU356394.1	1	0
	SFV_SXA; KP143760.1	1	0
	SFV-6; L25422	1	1

* References are given for those FVs where experimental miRNA data are available.

**Table 3:** Homology of seed sequences of experimentally identified BFV-Riems and simian FV of African green monkey SFV_agm_) miRNAs to known miRNAs of other species.

miRNA Name	Human and Bovine miRNA with Seed Identity
SFVagm -S2-5p	hsa-miR-28-5p, hsa-miR-3139, hsa-miR-708-5p
SFVagm -S3-5p	hsa-miR-4739, hsa-miR-4756-5p, hsa-miR-1321
SFVagm -S4-3p	hsa-miR-155-5p
SFVagm -S6-3p	hsa-miR-132-3p, hsa-miR-212-3p
SFVagm -S7-5p	hsa-miR-3154
BFV Riems miR-BF1-3p	bta-miR-125a, bta-miR-125b, bta-miR-670
BFV Riems miR-BF1-5p	bta-miR-3957
BFV Riems miR-BF2-5p	bta-miR-199a-3p
